# Pre-cooling for endurance exercise performance in the heat: a systematic review

**DOI:** 10.1186/1741-7015-10-166

**Published:** 2012-12-18

**Authors:** Paul R Jones, Christian Barton, Dylan Morrissey, Nicola Maffulli, Stephanie Hemmings

**Affiliations:** 1 Centre for Sports and Exercise Medicine, William Harvey Research Institute, Bart's and the London School of Medicine and Dentistry, Queen Mary University of London, Mile End Hospital, Bancroft Road, London E1 4DG, UK; 2 King's College London School of Medicine and Dentistry, King's College London, Guy's Campus, London SE1 9UL, UK

**Keywords:** Pacing, thermoregulation, internal cooling, cooling garment, cold water immersion, ice slurry ingestion

## Abstract

**Background:**

Endurance exercise capacity diminishes under hot environmental conditions. Time to exhaustion can be increased by lowering body temperature prior to exercise (pre-cooling). This systematic literature review synthesizes the current findings of the effects of pre-cooling on endurance exercise performance, providing guidance for clinical practice and further research.

**Methods:**

The MEDLINE, EMBASE, CINAHL, Web of Science and SPORTDiscus databases were searched in May 2012 for studies evaluating the effectiveness of pre-cooling to enhance endurance exercise performance in hot environmental conditions (≥ 28°C). Studies involving participants with increased susceptibility to heat strain, cooling during or between bouts of exercise, and protocols where aerobic endurance was not the principle performance outcome were excluded. Potential publications were assessed by two independent reviewers for inclusion and quality. Means and standard deviations of exercise performance variables were extracted or sought from original authors to enable effect size calculations.

**Results:**

In all, 13 studies were identified. The majority of studies contained low participant numbers and/or absence of sample size calculations. Six studies used cold water immersion, four crushed ice ingestion and three cooling garments. The remaining study utilized mixed methods. Large heterogeneity in methodological design and exercise protocols was identified. Effect size calculations indicated moderate evidence that cold water immersion effectively improved endurance performance, and limited evidence that ice slurry ingestion improved performance. Cooling garments were ineffective. Most studies failed to document or report adverse events. Low participant numbers in each study limited the statistical power of certain reported trends and lack of blinding could potentially have introduced either participant or researcher bias in some studies.

**Conclusions:**

Current evidence indicates cold water immersion may be the most effective method of pre-cooling to improve endurance performance in hot conditions, although practicality must be considered. Ice slurry ingestion appears to be the most promising practical alternative. Interestingly, cooling garments appear of limited efficacy, despite their frequent use. Mechanisms behind effective pre-cooling remain uncertain, and optimal protocols have yet to be established. Future research should focus on standardizing exercise performance protocols, recruiting larger participant numbers to enable direct comparisons of effectiveness and practicality for each method, and ensuring potential adverse events are evaluated.

## Background

Endurance exercise capacity has been reported to be diminished when exercising in hot environmental conditions, compared with normal and cold conditions [1-3]. A recent review evaluated the data of six International Association of Athletics Federations (IAAF) Gold Labeled Road Marathon races from 2001 to 2010 to determine which environmental factors have the largest impact on race performance [4]. The authors reported a median optimum environmental temperature of 6.2°C for men and 6.8°C for women. There was a consistent slowing of 0.03% for every 1°C increase in temperature above optimum and average performance decreases of -17.7% and -12.4% for men and women at +20°C above optimum. The authors concluded that temperature is the main environmental factor influencing marathon performance. Hot environmental temperatures also limit cycling performance. Peiffer and Abbiss [3] investigated cyclists performing a 40 km time trial in a heat chamber at different environmental temperatures. The authors reported a significantly lower mean power output for the participants at 37°C compared to at 17°C, 22°C and 27°C. A separate study reported that higher environmental temperatures reduce the time taken to reach volitional fatigue when cycling at a fixed intensity (70% maximal aerobic capacity (VO_2_max)). Mean time to volitional fatigue decreased by 30 minutes between trials performed at 21°C (81.2 min) and 31°C (51.6 min) [1]. Although not all endurance events follow a linear model of performance decline with increasing environmental temperature [5], it is apparent that hot environmental temperatures above an optimum impair endurance exercise performance.

Understanding the physiological basis as to why the capacity to perform endurance exercise is reduced in hot (≥ 28°C) environments is needed to develop interventions that may improve performance. It was previously postulated that exhaustion in hot conditions was a result of circulatory failure (a reduction in cardiac output and muscle blood flow) diminishing the drive for further exercise [6]. However, Nielsen *et al. *[7] found evidence to challenge this. In this study athletes exercised at 60% VO_2_max until exhaustion for 9 to 12 days in 40°C heat. The authors reported that exhaustion coincided with a core temperature of 39.7 ± 0.15°C. With acclimation, the athletes took progressively longer to reach this core temperature. No reduction in cardiac output was found at exhaustion and the authors concluded that high core temperature rather than circulatory failure was the limiting factor. However, thermoregulation and cardiovascular functioning are not separate entities and a number of physiological adaptations occurred with acclimation, such as earlier onset of the sweating response and improved cardiovascular efficiency, reducing cardiovascular strain and slowing the rate of rise of core body temperature, which likely contributed to the lower core body temperature at a given point of exercise reported in this study [7]. Another proposed hypothesis was that fatigue may arise from decreased substrate availability given that there is an observed increase in the rate of muscle glycogen utilization, and therefore depletion, when exercising in the heat, though this seems unlikely [3,8]. Febbraio *et al. *[8] reported that carbohydrate ingestion during cycling at 70% VO_2_max in 33°C heat produced no ergogenic effect compared to a sweet placebo, nor did the athletes' blood glucose fall below resting levels during the trial. They concluded that fatigue was related to thermoregulatory factors as opposed to decreased substrate availability.

Current hypotheses propose that the critical limiting factor for exercise performance in the heat is an elevated core body temperature, at which an athlete will have to reduce their exercise intensity or risk heat-related injury [9]. It is thought that pre-cooling in hot environments will improve endurance exercise performance by lowering an athlete's preliminary core body temperature, thereby increasing the margin between the initial core temperature and temperatures at which athletic performance is affected. A lower core body temperature at a given point of exercise has a similar effect to that which occurs with acclimation [7] and enables athletes to exercise at higher intensities during self-paced exercise (or for a longer duration during constant pace exercise). A consistent core temperature at voluntary fatigue has also been observed across fitness groups [10]. The higher environmental heat load in hot conditions augments the rate of rise in core body temperature, reducing the time taken for an athlete to reach their limiting temperature [11].

The hypothesized link between increased core temperature and reduced endurance exercise performance has led to the proposal and evaluation of a number of cooling methods prior to sports participation (that is, pre-cooling). It is thought that pre-cooling in hot environments will improve endurance exercise performance by lowering an athlete's preliminary core body temperature and increasing the margin between the initial core temperature and critical limiting core temperature at which athletic performance declines [12]. An athlete would therefore have a lower core body temperature at a given point of exercise, similar to the effect that occurs with acclimation reported in the Nielsen *et al. *study [7], enabling athletes to exercise harder for longer. Early pre-cooling studies evaluated the effectiveness of methods such as cold water baths and cooling fans, with positive outcomes for endurance exercise performance reported [13,14]. However, clinical application of these methods is made difficult by the need for transportation and/or installation of equipment and facilities needed.

The potential of pre-cooling to improve sporting performance led scientists at the Australian Institute of Sport (AIS) to develop a cooling jacket for in-competition athletes, constructed from neoprene and designed to be packed with ice, prior to the Atlanta Games 1996, as a more practical and convenient alternative to the cold water baths and cooling fans used in laboratory studies. Of the 43 surveyed after Atlanta, all athletes felt that the jackets made a positive contribution to their performance at the Games [15]. Since this practical innovation, other novel pre-cooling strategies have been proposed and investigated, such as ice slurry ingestion [16-19].

### Pre-cooling: theoretical mechanism of action

Different pre-cooling interventions are proposed to act via different mechanisms to reduce core body temperature and thus cool the body prior to exercise.

#### Cold water immersion

When immersed in water of an ambient temperature below the human thermoneutral zone in water (33 to 34°C), the human body will attempt to maintain its core temperature by reducing skin blood flow (vasoconstriction) [20]. Below this thermoneutral zone, vasoconstriction in isolation is not sufficient to maintain core temperature, so metabolic heat production is increased. However, if the cold stimulus is of a sufficiently low temperature and applied for long enough, heat loss will exceed heat production, causing a reduction in core temperature and increasing heat storage capacity [21].

#### Ice slurry ingestion

The phase change of solid ice (H_2_O) to liquid water requires a large transfer of heat energy into the system, known as the 'enthalpy of fusion (melting)' of ice. Merrick *et al. *[22] reported that cold modalities that undergo phase change caused lower skin surface and intramuscular temperatures than modalities that do not undergo phase change. Therefore, when ice slurry is ingested, heat energy is transferred into the slurry mix from the surrounding tissues, rather than stored in the body, reducing the core temperature. A study investigating intravenous cooling in swine reported that ice slurry (-1°C to 0°C) cooled brain temperature more rapidly and effectively than chilled saline (0°C to 1°C) [23], which suggests that ice slurry may potentially be effective as a pre-exercise pre-cooling modality.

#### Cooling garment

Cooling garments primarily reduce skin temperature. Common strategies include wearing a vest that covers the torso with pockets for ice packs (ice vest) [24-26], or wearing a waist-length polyester blend shell with sleeves and a hood that has a phase change material sewn in (cooling jacket) [17,27]. Kay *et al. *[28] suggested that lowering skin temperature without a concomitant reduction in core body temperature prior to exercise, increasing the thermal gradient between core and skin, afforded participants a lower core temperature during exercise due to increased core to skin heat loss.

Although there is a significant body of work regarding pre-cooling and its effects on athletic performance, the literature concerning pre-cooling for endurance exercise performance has yet to be reviewed systematically and it is yet to be established which pre-cooling modality or mechanism of body cooling is the most effective. Two reviews provide comprehensive descriptions of pre-cooling and its application to sports performance [29,30]. However, neither combined available data to systematically analyze or compare different pre-cooling strategies. The conclusions drawn are therefore more open to bias than those of a systematic review and comparisons of methods subjective. Furthermore, both reviews were published before more recent pre-cooling strategies such as ice slurry ingestion had been investigated and reported on. Therefore, a more up-to-date evidence-based review, less open to bias is warranted.

Recently, Ranalli *et al. *[31], evaluating the effect of body cooling on aerobic and anaerobic exercise performance, concluded that pre-cooling conferred limited benefit to intermittent or anaerobic exercise performance. For the 'aerobic' section of the review, they included nine studies, yet only two of these studies evaluated cooling prior to exercise (pre-cooling). Considering practical limitations to cooling during competition for many sports, and the number of additional studies evaluating the effects of pre-cooling on endurance exercise performance in the literature, a systematic review of all studies where participants were cooled prior to exercise is required. Therefore, the aims of this systematic review were to (i) summarize the effectiveness of different pre-cooling procedures to improve endurance exercise performance by comparing, critiquing and combining results from each study; (ii) enable evidence-based decisions on appropriate pre-cooling athlete management to be made; and (iii) provide guidance for future research evaluating the efficacy of pre-cooling strategies which aim to enhance endurance exercise performance.

## Methods

### Inclusion and exclusion criteria

Repeated measures crossover studies and randomized controlled trials comparing a pre-cooling method(s) to control or no intervention in healthy adults were considered for inclusion. The pre-cooling method could be any that cooled a participant prior to commencing an endurance exercise protocol or event. A measure of aerobic endurance was required to be one of the outcome measures in each study. The ambient environmental temperature during the performance trials had to be at or above the human thermoneutral zone of environmental temperatures (≥ 28°C) [32,33].

Unpublished studies, case series studies, non-peer-reviewed publications, studies not involving humans, reviews, letters, opinion articles, articles and abstracts not in English were excluded. Studies that included participants with pathological conditions known to increase susceptibility to heat strain, such as spinal cord injury [34], were also excluded, as were studies that attempted to cool participants during exercise, those that used intermittent or team-based sport exercise protocols or protocols that primarily stressed the anaerobic energy pathway. Unpublished research was not sought. Although this may potentially lead to publication bias [35], it was deemed impractical to identify all unpublished work on pre-cooling and endurance exercise performance from all authors and institutions around the world.

### Search strategy

The following databases were searched in May 2012 (week 4): MEDLINE (Ovid Web, 1948 to 2012 and Medline In-process and Other Non-Indexed Citations), EMBASE (1974 to 2012), CINAHL (1981 to 2012), Web of Science (1899 to 2012) and SPORTDiscus. Key terms used in the search strategy and results of the search are shown in Table 1. Reference lists and lists of citing articles were searched to ensure that no relevant studies had been missed by the search strategy. No additional papers were identified.

**Table 1:** Search strategy and results from each included database

**Search term/No**.	MEDLINE	EMBASE	CINAHL	Web of Science	SPORTDiscus
1. Exercise	195,255	277,329	61,991	83,075	165,077
2. Exercising	6,474	7,981	1,441	224,807	3,909
3. Endurance	21,579	23,146	5,353	24,920	19,791
4. Performance	456,095	712,833	59,983	1,652,852	130,350
5. Pace	9,687	12,271	2,880	46,122	5,847
6. Pacing	30,907	35,549	6,433	174,740	12,163
7. Sport	12,294	58,671	4,820	56,118	650,712
8. Sports	45,270	41,390	17,268	As above	650,460
9. Sporting	2,332	3,711	2,719	As above	101,200
10. Aerobic	47,428	68,495	6,143	59,356	21,497
11. OR/terms 1 to 10	74,560	1,114,664	137,399	2,094,266	871,407
12. Pre-cool	7	10	1	47	3
13. Pre-cool	7	13	0	494	2
14. Pre-cooling	115	193	18	544	50
15. Pre-cooling	51	94	7	473	48
16. Pre-cooled	144	141	3	258	4
17. Pre-cooled	76	75	0	473	1
18. Cool	4,854	6,454	905	214,304	2,906
19. Cooled	7,789	9,630	231	204,690	200
20. Cooling	22,183	31,464	940	As above	1,145
21. OR/terms 12 to 20	31,903	43,280	1,919	214,676	4,022
22. 11 AND 21	2,489	3,606	326	25,367	2,233
23. Limit 22 to English language	2,373	3,358	322	1,089^a^	2,147

^a^Search also refined by appropriate categories: sports science; physiology; public, environmental and occupational health; cardiac cardiovascular systems, respiratory system.

### Review process

All titles and abstracts were downloaded into EndNote X4 (Thomson Reuters, Philadelphia, PA, USA) giving a set of 9922 citations. The set was crossreferenced and any duplicates were deleted, leaving a total of 4454 citations. Each title and abstract were evaluated for potential inclusion by two independent reviewers (PRJ and CB) using a checklist developed from the inclusion/exclusion criteria outlined above. If insufficient information was contained in the title and abstract to make a decision on a study, it was retained until the full text could be obtained for evaluation. Any disagreements regarding studies were resolved by a consensus meeting between the two reviewers, and a third reviewer (DM) was available if necessary.

### Methodological quality assessment

Quality assessment was performed using the Physiotherapy Evidence Database (PEDro) Scale, which is a valid measure of the methodological quality of clinical trials [36]. Each study is rated according to ten separate criteria on the PEDro scale that assess a study's internal validity and statistical reporting, then totaled to give a score out of 10. An additional criterion, 'sample size calculation', was included in the quality assessment as the authors felt it to be an important component of study methodology. This criterion did not contribute to the PEDro score. Two reviewers (PRJ and CB) applied the PEDro scale to each included study independently, and any scoring discrepancies were resolved through a consensus meeting, with a third reviewer (DM) available if necessary. Studies were considered high quality if PEDro scores were greater than 6, and low quality if 6 and below.

### Statistical analysis and data synthesis

Means and standard deviations for all continuous data were extracted and effect sizes (Cohen's d) (with 95% CIs) calculated to allow comparison between the results of each study. Data were pooled using RevMan for Mac version 5.1.2 (The Nordic Cochrane Centre, Copenhagen, Denmark). If inadequate data were available from original studies to complete effect size calculations, attempts were made via email to contact the study's corresponding author for the required data. The presence of publication bias was determined by evaluating funnel plot asymmetry graphically [37,38].

Definitions for 'levels of evidence' were guided by recommendations made by van Tulder *et al. *[39] and are as given below:

'Strong evidence' = consistent findings among multiple high quality randomized controlled trials.

'Moderate evidence' = consistent findings among multiple low quality randomized controlled trials and/or non-randomized controlled trials, or one high quality randomized controlled trial.

'Limited evidence' = findings from one low quality randomized or non-randomized controlled trial.

'Conflicting evidence' = inconsistent findings among multiple trials.

## Results

**Figure 1:**
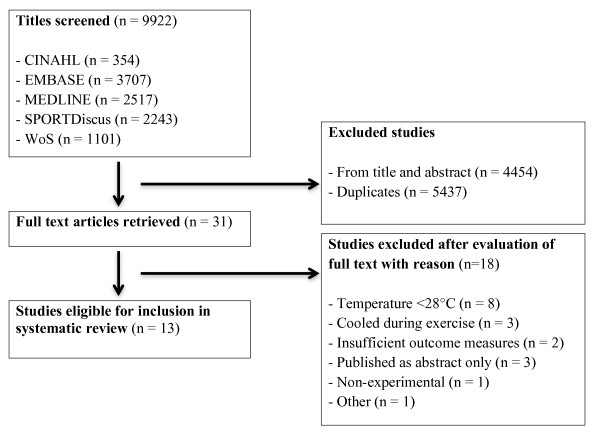
**QUOROM (for 'Quality of Reporting of Meta-analyses' using standards developed by the QUOROM group) flow diagram, summarizing study selection for inclusion**.

Following the search, 13 studies were deemed appropriate for inclusion (Figure 1). Table 2 shows the participant characteristics and investigation protocol for each included study.

**Table 2:** Investigation protocol for each included study.

Authors	Participant characteristics^a^	Exercise duration (min)	Cooling mode^b^	Cooling duration (min)	Environmental conditions		Performance task	Core temperature measurement	Outcome measure
								
					Temperature (°C)	Humidity (%)			
Arngrïmsson *et al*., 2004 [24]	9 male, 8 female; trained	15.6 to 22.8^c^	G	38	32	50	Running	Rectal	Time to complete 5 km
Booth *et al*., 1997 [42]	5 male, 3 female; trained	30	W	60	32	62	Running	Rectal	Distance completed in a 30-minute test at self-controlled pace
Cotter *et al*., 2001 [25]	9 male; untrained	35	G ± LC	45	35	60	Cycling	Rectal	Mean power output (W/kg) during 15 minutes at self-selected pace
Duffield *et al*., 2010 [41]	8 male; trained	40	W	20^d^	33	50	Cycling	Rectal	Mean power output (W) during 40-minute time trial
Gonzalez-Alonso *et al*., 1999 [40]	7 male; trained	42 to 66^c^	W	30	40	19	Cycling	Esophageal	Time to volitional fatigue at 60% VO_2_max
Hasegawa *et al*., 2006 [43]	9 male; untrained	2.5 to 8.0^e, f^	W/D/W + D	30	32	80	Cycling	Rectal	Time to volitional fatigue at 80% VO_2_max
Ihsan *et al*., 2010 [16]	7 male; trained	70 to 103^c^	I	30	30	75	Cycling	Gastrointestinal	Time to complete 40 km; mean power output (W)
Kay *et al*., 1999 [28]	7 male; trained	30	W	58.6	31	60	Cycling	Rectal	Distance completed in a 30-minute test at self-controlled pace
Quod *et al*., 2008 [27]	6 male; trained	40	G/W + G	40/70^g^	34	41	Cycling	Rectal	Time to complete a fixed amount of work (kJ); mean power output (W)
Ross *et al*., 2011 [17]	11 male; trained	76 to 123^c^	W + G/I^h^	30	32 to 35	50 to 60	Cycling	Rectal	Time to complete 23 km; mean power output (W)
Siegel *et al*., 2010 [18]	10 male; untrained	40.7 to 50.2^c^	I	30	34	55	Running	Rectal	Time to volitional fatigue at first ventilatory threshold
Siegel *et al*., 2012 [19]	8 male; untrained	46.7 to 56.8^c^	I/W	30	34	52	Running	Rectal	Time to volitional fatigue at first ventilatory threshold
Ückert and Joch, 2007 [26]	20 male; trained	26.9 to 32.5^c^	G/WU	20	30 to 32	50	Running	Tympanic	Time to volitional fatigue during an incremental treadmill test

^a^Described as moderately trained to well trained in sports with high endurance components by the study authors.

^b^W = cold water immersion, G = cooling garment, D = cool water drink, WU = warm-up, I = ice slurry ingestion, LC = leg cooling.

^c^Mean group time.

^d^Cooling was maintained during subsequent warm-up by application of cool gel packs to hamstrings and quadriceps.

^e^Preceded by 10 minutes at 50% and 30 minutes at 70% VO_2_max.

^f^Preceded by 60 minutes at 60% VO_2_max.

^g^30 minutes cold water immersion, followed by 40 minutes wearing a cooling garment.

^h^While applying iced towels.

VO_2_max = maximal aerobic capacity.

### Quality assessment of included studies

Of the 13 studies included in the review, 8 studies attained a PEDro score of 6/10 [16-19,26,27,40,41], 4 attained a score of 5/10 [24,25,42,43], and 1 study received a score of 4/10 (Table 3) [25]. Sample size calculations were not performed by any of the reviewed studies.

**Table 3:** Physiotherapy Evidence Database (PEDro) scale scores for each study

	Authors, year and reference												
	
Factor	**Arngrïmsson *et al*., 2004 **[**24**]	**Booth *et al*., 1997 **[**42**]	**Cotter *et al*., 2001 **[**25**]	**Duffield *et al*., 2010 **[**41**]	**Gonzalez-Alonso *et al*., 1999 **[**40**]	**Hasegawa *et al*., 2006 **[**43**]	**Ihsan *et al*., 2010 **[**16**]	**Kay *et al*., 1999 **[**28**]	**Quod *et al*., 2008 **[**27**]	**Ross *et al*., 2011 **[**17**]	**Siegel *et al*., 2010 **[**18**]	**Siegel *et al*., 2012 **[**19**]	**Ückert and Joch, 2007 **[**26**]
Eligibility criteria were specified (not scored)	0	0	0	0	0	0	0	0	0	0	0	0	0
Subjects were randomly allocated to groups	0	0	0	1	1	0	1	0	1	1	1	1	1
Allocation was concealed	0	0	0	0	0	0	0	0	0	0	0	0	0
Groups were similar at baseline	1	1	1	1	1	1	1	1	1	1	1	1	1
Blinding of subjects	0	0	0	0	0	0	0	0	0	0	0	0	0
Blinding of intervention administrators	0	0	0	0	0	0	0	0	0	0	0	0	0
Blinding of assessors	0	0	0	0	0	0	0	0	0	0	0	0	0
Outcome measure obtained from ≥ 85% subjects	1	1	1	1	1	1	1	1	1	1	1	1	1
All subjects received intervention/intention to treat analysis	1	1	0	1	1	1	1	1	1	1	1	1	1
Between group statistical comparisons reported	1	1	1	1	1	1	1	1	1	1	1	1	1
Between-group variability reported	1	1	1	1	1	1	1	1	1	1	1	1	1
PEDro score (out of 10)	5	5	4	6	6	5	6	5	6	6	6	6	6
Sample size calculation performed	0	0	0	0	0	0	0	0	0	0	0	0	0

### Additional data and publication bias

Corresponding authors of two additional studies eligible for review were contacted via email to request additional data necessary for inclusion in the review [44,45]. The required data had not been supplied at the time of going to press. A symmetrical funnel plot indicated the absence of publication bias [37,38].

### Effectiveness of different pre-cooling modalities

#### Cold water immersion

**Figure 2:**
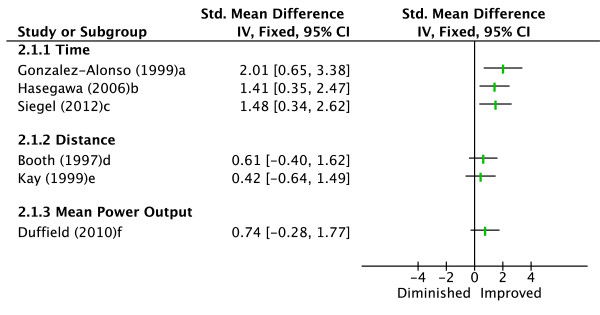
**Effect sizes (Cohen's d) for cold water immersion versus control**. Graph represents effect of intervention on exercise performance. ^a^Time to volitional fatigue running at 60% VO_2_max. ^b^Time to volitional fatigue cycling at 80% VO_2_max. ^c^Time to volitional fatigue at first ventilatory threshold. ^d^Distance run in 30 minutes at self-controlled pace. ^e^Distance cycled in 30 minutes at self-controlled pace. ^f^Mean power output during 40-minute cycling time trial.

Six studies evaluated the effectiveness of cold water immersion in enhancing endurance exercise performance compared to a control condition (see Figure 2) [19,28,40-43]. Performance measures evaluated included time to volitional fatigue exercising at a fixed exercise intensity [19,40,43], distance completed in a 30-minute self-controlled exercise test [28,42], and mean power output (MPO) over a 40-minute cycling time trial [41]. Three studies showed improved performance compared to a control condition (d = 2.01, 1.41 and 1.48 respectively) [19,40,43]. Consistent with significant findings, the remaining three studies showed a trend for cold water immersion to improve performance, though this was not statistically significant (d = 0.61, 0.42 and 0.74 respectively) [28,41,42]. Therefore, moderate evidence is indicated for the effectiveness of cold water immersion to improve endurance exercise performance in hot environments.

#### Ice slurry ingestion

**Figure 3:**
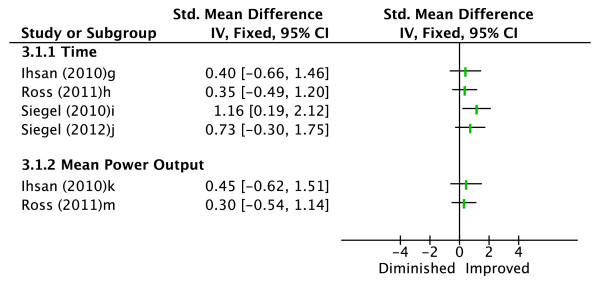
**Effect sizes (Cohen's d) for ice slurry ingestion versus control**. Graph represents effect of intervention on exercise performance. ^g^Time to cycle 40 km. ^h^Time to cycle 23 km. ^i^Time to volitional fatigue at first ventilatory threshold. ^j^Time to volitional fatigue at first ventilatory threshold. ^k^Mean power output cycling 40 km. ^m^Mean power output cycling 23 km.

Four studies evaluated the effectiveness of ingesting an ice slurry beverage in enhancing endurance exercise performance compared to a control condition (see Figure 3) [16-19]. The control condition was consumption of a volume of water equal to that of the ingested ice slurry in each study. Performance measures evaluated included time taken to cycle a set distance and MPO [16,17], and time to volitional fatigue at a fixed exercise intensity [18,19]. One study showed a statistically significant performance improvement in the pre-cooling condition (d = 1.16) [18]. All three remaining studies reported a trend towards improved performance in the pre-cooling condition for both time taken and MPO [16,17,19]. Therefore, limited evidence is indicated for the effectiveness of ice slurry ingestion to improve endurance exercise performance in hot environments.

#### Cooling garment

**Figure 4:**
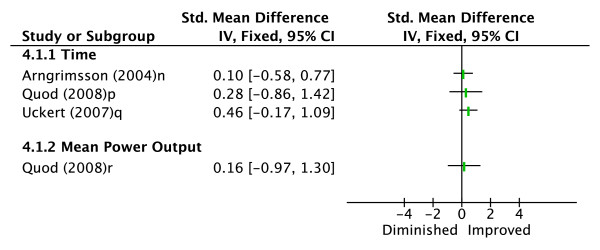
**Effect sizes (Cohen's d) for cooling garment versus control**. Graph represents effect of intervention on exercise performance. ^n^Time to run 5 km. ^p^Time to cycle a fixed amount of work. ^q^Time to volitional fatigue during an incremental treadmill run. ^r^Mean power output for duration of cycling time trial.

Three studies evaluated the effectiveness of a cooling garment in enhancing endurance exercise performance compared to a control condition (see Figure 4) [24,26,27]. Two studies [24,26] used an ice vest as their cooling garment, and the other used a cooling jacket covering the torso, arms, and head with a hood [27]. Performance measures evaluated included time taken to complete a 5 km run [24], time to volitional fatigue on an incremental treadmill test [26], and time taken to complete a fixed amount of work (kJ) and MPO while cycling [27]. There were no significant improvements in performance for any of the parameters measured, indicating moderate evidence that cooling garments are an ineffective pre-cooling intervention.

#### Mixed cooling methods

**Figure 5:**
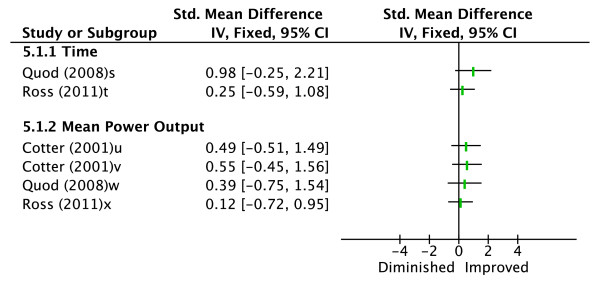
**Effect sizes (Cohen's d) for various mixed cooling methods versus control**. Graph represents effect of intervention on exercise performance. ^s^Time to cycle a fixed amount of work (cold water immersion + cooling garment). ^t^Time to cycle 23 km (cold water immersion + cooling garment). ^u^Mean power output during 15-minute cycling time trial (cold air + cooling garment + leg cooling). ^v^Mean power output during 15-minute cycling time trial (cold air + cooling garment + leg warming). ^w^Mean power output for duration of cycling time trial (cold water immersion + cooling garment). ^x^Mean power output cycling 23 km (cold water immersion + cooling garment).

Three studies evaluated the effectiveness of combined pre-cooling methods to a control condition (see Figure 5) [17,25,27]. Two studies pre-cooled athletes using cold water immersion followed by wearing a cooling jacket (torso, sleeves and hood) [17,27]. Performance measures evaluated in both studies were time taken to cycle a set distance and MPO. Although not statistically significant, Quod *et al. *[27] showed a trend towards the pre-cooling condition improving performance (d = 0.98, 0.39 for time taken and MPO respectively). Ross *et al. *[17] found no improvement. Cotter *et al. *[25] used two different, mixed methods pre-cooling procedures in their study. Subjects were cooled with an ice vest and cold air while their thighs were either kept warm or cooled using water-perfused cuffs. The performance measure evaluated was MPO. Both of these pre-cooling interventions showed a trend to performance improvement in the pre-cooled conditions (d = 0.49 and 0.55 in leg cooling and leg warming, respectively).

#### Comparison of pre-cooling methods

**Figure 6:**
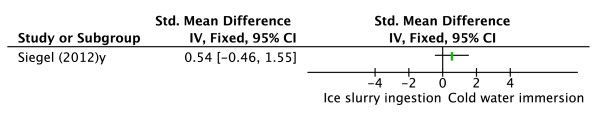
**Effect sizes (Cohen's d) for ice slurry ingestion versus cold water immersion**. Graph represents effect of intervention on exercise performance. ^y^Time to volitional fatigue at first ventilatory threshold.

One study evaluated the effectiveness of ice slurry ingestion in enhancing endurance exercise performance compared to cold water immersion (see Figure 6) [19]. There was no statistically significant difference between the two pre-cooling methods (d = 0.54), though there was a trend to cold water immersion being more effective. There is limited evidence that ice slurry ingestion is as effective at improving endurance exercise performance as cold water immersion.

## Discussion

The aim of the present systematic review was to summarize the effectiveness of different pre-cooling techniques to improve endurance exercise performance in hot (≥ 28°C) environmental conditions. A total of 13 studies contained sufficient data to complete effect size calculations [16-19,24-28,40-43]. Of the three individual pre-cooling methods identified, cold water immersion was the most effective, with moderate evidence supporting its ability to improve endurance exercise performance compared to control conditions. Additionally, limited evidence indicates that ingesting ice slurry prior to competition is also effective, and potentially a more practical alternative to cold water immersion. Wearing a cooling garment prior to endurance exercise is of limited benefit to subsequent endurance exercise performance. Of the combined pre-cooling procedures that improved performance, the most effective protocol involved a period of cold water immersion.

### Quality

Each included study used a repeated measures crossover design. However, methodological quality was varied with PEDro scores ranging from 4/10 to 6/10, indicating no high quality randomized controlled trials evaluating the effectiveness of pre-cooling to improve endurance exercise performance in the heat. Some studies did not randomize participant allocation, possibly introducing allocation bias [24,25,28,42,43]. All except four studies [18,19,25,43] used participants who were moderately to well trained (Table 4) in sports with high endurance components (cycling, triathlon and distance running), and within that only cycling and running exercise protocols were used, limiting the applicability of the findings to the broader, less well trained population. Lack of participant, investigator and outcome assessor blinding was consistent across all studies, likely due to practical difficulties. Consequently, some results could have been unintentionally biased, either by observer bias, such as encouraging participants in the pre-cooled group, or a placebo effect.

**Table 4:** Participant characteristics for each included study

Authors, year and reference	Participant characteristics
Arngrïmsson *et al*., 2004 [24]	Competitive collegiate and club middle/long distance runners
	Age men: 23.4 (4.4) years
	Age women: 22.1 (2.2) years
	Height men: 178.6 (4.4) cm
	Height women: 167.7 (5.5) cm
	Body mass men: 67.7 (4.2) kg
	Body mass women: 55.9 (4.3) kg
	Body fat men: 7.3 (2.0) %
	Body fat women: 17.8 (3.3) %
	Best 5 km run time men: 15.5 (0.8) min
	Best 5 km run time women: 17.9 (1.1) min
	VO_2_max men: 4.50 (0.31) l/min
	VO_2_max women: 3.24 (0.25) l/min
	Heat acclimatized
Booth *et al*., 1997 [42]^a^	Competitive runners from a local athletic club
	Age: 26.7 (1.7) years
	Height: 169.7 (4.0) cm
	Weight: 65.96 (2.87) kg
	Sum of eight skinfolds: 62.5 (9.7) mm
	Body surface area: 1.75 (0.06) m^2^
	Body fat: 15.8 (1.2) %
	HR_max_: 189.5 (2.8) beats/min
	VO_2_peak: 63.1 (0.1) ml/kg/min
	Non-heat acclimatized
Cotter *et al*., 2001 [25]	Habitually active, but were of lower average aerobic fitness than subjects used in previous studies on the effects of pre-cooling
	Age: 32.4 (3.6) years
	Height: 175.6 (6.9) cm
	Body mass: 80.9 (10.5) kg
	Body surface area: 1.96 (0.15) m^2^
	VO_2_peak: 51 (8) ml/min/kg
	Non-heat acclimatized
Duffield *et al*., 2010 [41]	Moderate to well trained cyclists of club and regional standard who trained multiple times a week, competing in regional competitions
	Age: 24.8 (3.3) years
	Height: 178.3 (8.0) cm
	Body mass: 76.1 (2.7) kg
	Sum of seven skinfolds: 54.4 (10.9) mm
	Lactate threshold: 221 (42) W
	Non-heat acclimatized
Gonzalez-Alonso *et al*., 1999 [40]*	Endurance trained
	Age: 28 (3) years
	Height: 187 (6) cm
	Body mass: 77.9 (6.4) kg
	HR_max_: 200 (9) beats/min
	VO_2_peak: 5.13 (0.30) l/min
	Non-heat acclimatized
Hasegawa *et al*., 2006 [43]*	Untrained
	Age: 21.8 (0.8) years
	Height: 1.72 (0.02) cm
	Body mass: 61.7 (2.1) kg
	Body fat: 15.1 (1.1) %
	VO_2_max: 48.5 (1.5) ml/kg/min
	Non-heat acclimatized
Ihsan *et al*., 2010 [16]	Endurance trained regularly competing in cycling or triathlon, cycling more than four sessions and > 150 km/week
	Age: 27.7 (3.1) years
	Height: 176.7 (5.8) cm
	Body mass: 81.38 (9.09) kg
	Non-heat acclimatized
Kay *et al*., 1999 [28]*	Moderately to well-trained and undertook bicycle riding, training and competition on a regular basis
	Age: 23.7 (2.1) years
	Height: 182 (3) cm
	Body mass: 76.1 (4.0) kg
	Sum of four skinfolds: 28.4 (2.3) mm
	Body surface area: 1.97 (0.06) m^2^
	HR_max_: 184 (3) beats/min
	VO_2_peak: 4.91 (0.25) l/min
	Non-heat acclimatized
Quod *et al*., 2008 [27]	Well trained male cyclists with 6 (5) years of experience
	Age: 28 (4) years
	Height: 182 (2) cm
	Body mass: 75.1 (3.2) kg
	Sum of seven skinfolds: 50 (11) mm
	VO_2_peak: 71.4 (3.2) ml/kg/min
	Maximum aerobic power: 384 (23) W
	Non-heat acclimatized
Ross *et al*., 2011 [17]	Well trained A-grade cyclists aged 18 to 35 years
	Age: 33 (5.1) years
	Body mass: 72.1 (5.5) kg
	Maximum aerobic power: 449 (26) W
	VO_2_peak: 71.6 (6.1) ml/kg/min
	Heat acclimatized
Siegel *et al*., 2010 [18]	Moderately active, participating in recreational sport
	Age: 28 (6) years
	Height: 178.9 (6.3) cm
	Body mass: 79.9 (11.2) kg
	Sum of nine skinfolds: 92.8 (41.4) mm
	VO_2_peak: 56.4 (4.7) ml/kg/min
	Non-heat acclimatized
Siegel *et al*., 2012 [19]	Moderately active, were partaking in recreational sport
	Age: 26 (4) years
	Height: 179.9 (6.7) cm
	Body mass: 78.1 (5.9) kg
	Sum of nine skinfolds: 87.3 (22.5) mm
	VO_2_peak: 54.2 (2.5) ml/kg/min
	Non-heat acclimatized
Ückert and Joch, 2007 [26]	Regularly practiced types of sport with high endurance and strength components at a high level for example, soccer, athletics
	Age: 25.6 (3.5) years
	Height: 183.4 (7.6) cm
	Weight: 77.9 (9.5) kg
	Non-heat acclimatized

All values are mean (± SD).

^a^Values in brackets are ± (SE).

HR = heart rate; VO_2_max = maximal aerobic capacity; VO_2_peak = peak oxygen uptake.

Participant numbers in each study were low, ranging from 6 [27] to 20 [26], limiting the validity of conclusions that can be drawn from the results. None of the reviewed studies performed sample size calculations, and therefore certain data trends could not be substantiated due to inadequate statistical power. There was a high level of methodological heterogeneity between studies, including: exercise performance protocol, pre-cooling duration, exercise duration and outcome measure, making comparison of studies and recommendations for enhancing sporting performance difficult. This was further compounded by the absence of comparisons between the three main individual pre-cooling maneuvers (cold water immersion, cooling garment and ice slurry ingestion) in all but one study [19]. Therefore, the relative efficacy and practicality of one pre-cooling method to another could not be made. In one study, subjects exercised at 60% VO_2_max for 60 minutes followed by an effort at 80% VO_2_max to volitional fatigue [43]. However, mean performance time ± standard error were only reported for the short effort at 80% VO_2_max to fatigue. This is likely to have inflated the effect size compared to other studies.

### Cold water immersion

Moderate evidence currently exists to support the use of cold water immersion as a pre-cooling intervention to improve endurance exercise performance in the heat. Three studies showed a significant performance improvement in the pre-cooled compared to control condition [19,40,43], with the remaining three studies showing a positive trend to improved performance [28,41,42]. In each of the immersion studies there was a significant reduction in core temperature compared to control at some point during the exercise protocol. Additionally, the rate of heat storage was greater in three of the four studies that reported this variable [19,28,42], conferring a greater margin for metabolic load during exercise in the pre-cooling condition. Gonzalez-Alonso *et al. *[40] reported that rate of heat storage was equal between both conditions. However, as the pre-cooled group commenced exercise with a core temperature 1.5°C lower than the control condition, their total heat storage capacity was greater. Although not conclusive evidence of a precise mechanism, it seems that pre-cooling using cold water immersion could possibly improve performance by reducing core temperature prior to exercise, or blunting the rate of rise in core temperature during exercise, increasing heat storage capacity and enabling athletes to perform at a greater relative intensity or for a greater duration [29].

Despite a more rapid reduction in core temperature with water immersion compared with traditional cold air exposure [46], the required length of pre-cooling remains significant (30 to 60 minutes) [29,30]. Marino and Booth [21], in one of the first studies investigating the potential use of pre-cooling via cold water immersion prior to endurance exercise, reduced core temperature by gradually reducing the temperature of the immersion bath over a 60-minute period. This was to avoid the potentially detrimental cold stress responses that had previously been seen with cold air exposure, such as shivering [29]. Such a regimented technique, which also precludes a concomitant warm-up, is limited in its practicality in an elite sports setting immediately prior to athletic competition, in addition to other logistical issues such as expense, transportation of equipment, and access to such a large volume of water and electricity in the field.

### Ice slurry ingestion

Limited evidence currently exists to support the use of ice slurry as a pre-cooling intervention to improve endurance exercise performance in the heat. One study [18] showed a significant performance improvement in the ice slurry ingestion pre-cooled compared to the control condition and the three remaining studies showed a positive trend to improved performance [16,17,19]. Each study reported that core temperature was significantly lower in the pre-cooling condition than control after the cooling intervention and prior to the start of the exercise task, increasing heat storage capacity. Alternatively, the participants' lower core body temperatures prior to exercise may have enabled them to select a faster pacing strategy by influencing central regulation of exercise intensity [47].

Two studies [18,19] reported that the pre-cooled group exhibited a significantly higher core temperature at exhaustion. The authors suggest that this could be due to the generation of higher metabolic heat loads as a result of either a direct cooling effect on the brain, or an effect on core temperature afferent nerves [48], altering perception of effort and increasing time to exhaustion. Increased core temperature above normal tolerable limits is an important safety consideration and may be detrimental to athlete health, increasing the risk of heat-related illness, and is something that requires attention in future studies.

Ice slurry ingestion offers a number of practical benefits over cold water immersion, as it is not subject to the same logistical restrictions. The ice slurry can be produced using a commercially available machine or simply freezing and part-thawing sports drinks prior to the event, and transporting them in a cool box. This is particularly useful at events where there is no provision for electrical equipment, or where transportation is an issue. Pre-cooling athletes in this way is quick and simple. The amount of ice slurry required to achieve effective cooling is low and similar in volume to pre-exercise fluid hydration protocols, ranging from 6.8 g/kg [16] to 14 g/kg [17] of body mass. In each reviewed study, the volume of ice slurry was administered over a 30-minute period at a standardized rate that ranged from 5 [18,19] to 15 minutes [17]. Although not yet investigated, there is the potential that ice slurry ingestion could enable athletes to warm-up during cooling, making it much more time efficient than cold water immersion. In addition to providing a greater cooling effect than cold water alone [23], a much smaller volume is required to produce this response, reducing the potential for detrimental effects that the ingestion of large volumes of fluid may have. As well as cooling athletes, the ice slurry can be used to hydrate athletes too so that combined fluid and slurry ingestion is not necessary.

### Cooling garments

None of the studies showed a significant improvement of wearing a cooling garment on subsequent exercise performance [24,26,27]. This likely resulted from the lack of effect on core body temperature. In two studies [26,27], despite the pre-cooling groups having significantly lower skin temperatures while wearing the cooling garment, core temperature was not significantly lower at any time point during either pre-cooling or subsequent exercise. Arngrïmsson *et al. *[24] reported significantly lower rectal temperatures in the pre-cooling group for the last 18 minutes of the warm-up and first 3.2 km of the running exercise task compared to the control group. However, this effect was not strong enough to have caused a significant improvement in performance and may have resulted from the high rectal temperatures, and therefore reduced heat storage capacity, at the start of the performance task in both the cooling garment (38.0°C) and control group (38.2°C) compared to all other studies that reported rectal temperature at the onset of the exercise task [18,19,25,27,28,41-43].

Kay *et al. *[28] suggested a reduction in core body temperature achieved through lowering skin temperature, effecting heat loss from core to skin. This is the mechanism by which cooling garments are believed to act to cool athletes prior to exercise. However, cooling in Kay *et al.'s *[28] study was achieved via whole-body cold water immersion, which likely provided a greater cooling stimulus than cooling garments, especially at peripheral areas of the body. This could explain why cooling garments were found to have little effect on core body temperature in the present study. One study reported than the application of a cooling garment reduced skin blood flow across the body by stimulating vasoconstriction, preventing efficient heat transfer between the skin and the cooling garment. Core body temperature of subjects remained unaltered, likely from the redistribution of blood to the core [44]. If the hypothesis that a critical core temperature limits exercise performance in the heat is correct, then, by failing to reduce core body temperature cooling garments were unable to improve endurance exercise performance.

### Mixed methods

Some studies combined more than one pre-cooling intervention to cool participants prior to the exercise component of the trial. Two studies immersed subjects in cold water, followed by a period wearing a cooling garment [17,27]. Quod *et al. *[27] reported a significant decrease in core body temperature prior to exercise, likely as a result of an 'afterdrop' effect [49]; that is, a continued fall in core temperature after the initial hypothermic exposure, as opposed to any further cooling effect of the garment. Indeed, the same study reported that wearing the cooling garment alone failed to reduce core temperature compared to control. Exercise performance was significantly better than control and cooling garment conditions. Conversely, Ross *et al. *[17] reported that, despite a significantly lower core body temperature after cooling and throughout warm-up compared to controls, there was no improvement in performance. The authors suggest that the larger cooling response in the combined condition may have led the athletes to select poorer pacing strategies. An alternative explanation could be that the cold water immersion protocol used may have been too abrupt compared to that used in other studies [21], and may therefore have elicited a cold stress response that was detrimental to performance, similar to that reported for cold air exposure [29].

A combination of cold air and a cooling garment, with or without thigh cooling, showed trends to improved performance in both conditions compared to control in one study [25]. Both pre-cooling groups had a lower core temperature after pre-cooling, and power output was significantly greater compared to controls during the 15-minute performance trial. There was no difference in power output between the two cooling conditions. It is difficult to determine whether the cooling garment conferred any additional benefits than have been shown to be conferred by cold air cooling alone [12,13,50]. In practice, cold air cooling has a number of logistical limitations including equipment transport and cost, the significant time required to adequately cool athletes, and a noted cold stress response that can impair exercise performance [29].

### Limitations and future research

There is a high level of heterogeneity in study design examining the effectiveness of pre-cooling strategies, and optimal cooling protocols have yet to be established. Variables such as cooling duration and time between pre-cooling and commencing exercise are likely to exert considerable influence on study outcomes and require greater attention. Once repeatable pre-cooling protocols have been identified for each individual modality, then more reliable comparisons of effectiveness can be made between modalities. One study directly compared ice slurry ingestion to cold water immersion and found it to be similarly effective at improving performance (Figure 6) [19]. As potentially the cheaper, more practical strategy, this result is encouraging and warrants further investigation of ice slurry ingestion in the field. Additionally, following Quod *et al.'s *study [27], it would be instructive to determine whether the combination of a cooling garment following cold water immersion confers any additional benefit compared to immersion only.

Hydration strategies employed, and reporting of these strategies was inconsistent (Table 5). Water ingestion, especially cool water, may lower core body temperature via a similar mechanism as ice slurry ingestion. Potentially, if control participants were permitted to drink cool water either before or throughout the exercise trial this may confound the effectiveness of the pre-cooling strategy. However, this is a difficult variable to control for and depends on the comparison being made. For example, studies investigating ice slurry ingestion used water ingestion of an equal volume as the control condition to determine that any improvements in performance were a result of the pre-cooling effect of ice slurry ingestion as opposed to the ergogenic effect of adequate hydration [51]. Interestingly, Siegel *et al. *found a greater effect of ice slurry ingestion on performance when compared to controls drinking cool fluid (4°C) [18] than when compared to controls drinking warmer fluid (37°C) [19], which suggests that cool water ingestion may not blunt the effectiveness of pre-cooling as much as expected. However, more consistent hydration protocols will enable greater analysis of this relationship. Hasegawa *et al. *[43] reported that continuous cool water ingestion during exercise following cold water immersion significantly improved performance compared to cold water immersion alone and negated the rise in core body temperature towards the end of the performance protocol. The authors attributed this to increased evaporative sweat loss, sweat efficiency and decreased heat strain in the continuous water ingestion group. This finding suggests that the benefits of pre-cooling may be augmented by maintaining hydration during exercise. Ice slurry ingestion acts to pre-cool athletes and could also be used to maintain cooling and hydration during exercise. Therefore, a comparison of combined cold water immersion and water beverage with continuous ice slurry ingestion is warranted.

**Table 5:** Hydration practices for each included study

Authors, year and reference	Hydration practice
Arngrïmsson *et al*., 2004 [24]	Pre-test: instructed to drink water and other non-caffeinated beverages liberally
	During the warm-up: water *ad libitum*. Tap temperature. Amount was recorded and repeated for the second condition.
	Not reported/performed during exercise
Booth *et al*., 1997 [42]	During exercise trial: water *ad libitum*
Cotter *et al*., 2001 [25]	Pre-test: instructed to drink at least 15 ml/kg BM 2 to 3 h before arrival at laboratory
	During the warm-up: water *ad libitum *after warm-up and before exercise trial
	Not reported/performed during exercise
Duffield *et al*., 2010 [41]	Pre-test: 500 ml water 60 min before arrival at the laboratory
	Not reported/performed during exercise
Gonzalez-Alonso *et al*., 1999 [40]	Pre-test: 200 to 300 ml with breakfast
	Not reported/performed during exercise
Hasegawa *et al*., 2006 [43]	Pre-test: 500 ml 2 h before the trial
	Immersion: no fluid ingestion
	Immersion + water ingestion: water (14 to 16°C) every 5 min during exercise equal to volume sweat loss in sweat test performed at a prior visit to laboratory
	Water ingestion: water (14 to 16°C) every 5 min during exercise equal to volume sweat loss in sweat test performed at a prior visit to laboratory
	Control: no fluid ingestion
Ihsan *et al*., 2010 [16]	Pre-test: adequate hydration was strongly encouraged before testing
	Pre-cooling: 6.8 g/kg BM ice slurry in 150 to 200 g aliquots in intervals of 8 to 10 minutes over a period of 30 minutes (1.4 ± 1.1°C)
	Control: 6.8 g/kg BM tap water slurry in 150 to 200 g aliquots in intervals of 8 to 10 minutes over a period of 30 minutes (26.8 ± 1.3°C)
	During exercise trial: 100 ml water (26.8 ± 1.3°C) at four intervals
Kay *et al*., 1999 [28]	During exercise trial: water *ad libitum*
Quod *et al*., 2008 [27]	Pre-test: 250 ml sport drink diluted to half the manufacturer's recommended strength
	During exercise trial: 250 ml sport drink diluted to half the manufacturer's recommended strength
Ross *et al*., 2011 [17]	Pre-test: water (4°C) *ad libitum *throughout heat stabilization and warm-up
	Pre-cooling: 14 g/kg BM ice slurry in two 7 g/kg BM boluses 15 minutes apart
	Control: water (4°C) *ad libitum*
	During exercise trial: subjects were provided with 350 ml of a 6% carbohydrate-electrolyte drink at 12.5 and 37.5 km to consume *ad libitum *for the next km (drinks left out in heat temperature to simulate race conditions)
Siegel *et al*., 2010 [18]	Pre-test: instructed to drink at least 2 l fluid the day before the trial, and 400 ml during the meal consumed before the trial
	Pre-cooling: 7.5 g/kg BM ice slurry (-1°C) with 5% carbohydrate in 1.25 g/kg BM aliquots every 5 minutes over a period of 30 minutes
	Control: 7.5 g/kg BM water (4°C) with 5% carbohydrate in 1.25 g/kg BM aliquots every 5 minutes over a period of 30 minutes
	Not reported/performed during exercise
Siegel *et al*., 2012 [19]	Pre-test: instructed to drink at least 2 l fluid the day before the trial, and 400 ml during the meal consumed before the trial
	Pre-cooling: 7.5 g/kg BM ice slurry (-1°C) with 5% carbohydrate in 1.25 g/kg BM aliquots every 5 minutes over a period of 30 minutes
	Immersion: 7.5 g/kg BM water (37°C) with 5% carbohydrate in 1.25 g/kg BM aliquots every 5 minutes over a period of 30 minutes
	Control: 7.5 g/kg BM water (37°C) with 5% carbohydrate in 1.25 g/kg BM aliquots every 5 minutes over a period of 30 minutes
	Not reported/performed during exercise
Ückert and Joch, 2007 [26]	Pre-test: avoid fluid for 3 h before start of test
	Not reported/performed during exercise

BM = body mass.

The level of fitness of participants varied across studies as did the consistency of reporting of fitness and experience of endurance exercise (Table 4). It is difficult, therefore, to determine whether more experienced or less experienced athletes would benefit more from pre-cooling. Furthermore, those who are less experienced are likely to be less accurate when anticipating a required pacing strategy to complete a given exercise trial [47]. By lowering core body temperature using exogenous means, participants may perceive their level of exertion to be lower than their body's thermal load should dictate, that is, a discrepancy between their perceived and actual homeostatic state, which could cause them to develop heat illness due to the masking of thermal strain. This is acknowledged in the two studies that reported participants to have an elevated core body temperature at volitional fatigue [18,19]. Notably, these studies used untrained participants so it is possible that more experienced athletes may be better attuned to their physiological limits and hence less at risk of heat illness, but this is speculative and warrants further investigation before ice slurry ingestion can be recommended.

Given the lack of blinding of participants and researchers in the reviewed studies, a placebo effect cannot be excluded from having influenced results. Future studies should consider introducing a separate, placebo-controlled group, and participant and assessor blinding to improve methodological validity. The placebo-control could, for example, use menthol to provide a cooling sensation for participants without causing an actual change in temperature [52].

Each study included in this review was limited by low participant numbers. It was therefore difficult to determine whether certain reported trends or lack thereof were the result of the studies being underpowered. *A priori *power calculations should be performed to increase the statistical significance of any trends reported in the results. Study participants were predominantly male, therefore the findings of this review may not be applicable to females, especially because certain anthropometric and hormonal differences, including stage of the menstrual cycle [53] and body composition [54], can affect thermoregulation under heat stress. It should be acknowledged that the majority were performed with the intention of applying the findings to highly trained athletes, and that recruiting large numbers of such compliant volunteers is difficult. However, the inclusion of larger sample sizes and inclusion of similar proportions of female and male participants in future research will allow both improved external validity for broader populations and between sex comparisons to be made.

Laboratory studies grant assessors strict control of certain variables, such as the environmental conditions under which exercise is performed, which is necessary with preliminary studies to establish intervention efficacy and optimal protocols. However, future studies of pre-cooling should focus on real-world testing to determine whether the promising laboratory findings translate to tangible performance gains in the field. This is also important to evaluate the practicality of each method of pre-cooling during competition.

There was also a lack of safety or adverse event reporting. It remains unknown what effect increased heat storage capacity may have on other bodily systems other than those directly involved in thermoregulation. Therefore, until these can be elucidated, it would be prudent for future research to include consideration of athlete safety, as this will be of primary concern to coaches and athletes alike, given the physiologically stressful environment in which they will be competing.

### Practicality and recommendations

Although consistently the most effective method of pre-cooling and enhancing endurance exercise performance in the heat, cold water immersion has limited practicality in sporting settings due to expense, transportation issues, difficulty accessing large volumes of water and time required to achieve a reduction in core body temperature. Ice slurry ingestion is a relatively cheap and much more practical alternative to whole body immersion and effectively lowers core body temperature, approaching the improvements in performance seen with immersion. Additionally, there is currently limited evidence from one study that indicates the effectiveness of ice slurry ingestion and cold water immersion are comparable [19]. However, safety concerns raised by two studies that reported a raised core body temperature at volitional fatigue need to be addressed before its use can be recommended for competing athletes. Cooling garments failed to improve endurance exercise performance and therefore are of limited use in this regard.

## Conclusions

This systematic review suggests that pre-cooling procedures can improve endurance exercise performance in the heat, with the likely mechanism being reduced core body temperature prior to exercise, and subsequently increased heat storage capacity. Cold water immersion is the most effective method of pre-cooling, with moderate evidence to support its effectiveness. However, its limited practicality in many sporting settings must be considered. Ice slurry ingestion has shown good initial results, with limited evidence supporting its effectiveness, and may provide a more practical pre-performance option. However, larger studies with consistent protocols and further investigation of potential safety issues associated with altered levels of perceived exertion are required before its use can be recommended. Cooling garments appear of limited efficacy, but this finding may be the result of suboptimal cooling protocols or inadequate study power. To date, most studies have focused on whether pre-cooling improves performance compared to no intervention, with only one study directly comparing individual modalities. Therefore, recommending one method over another to coaches and athletes is difficult. Further comparative research is required before best practice recommendations can be made.

## Competing interests

The authors declare that they have no competing interests.

## Authors' contributions

PRJ conceived the review topic. All authors assisted with the review design. PRJ and CB completed searching, quality assessment and data analysis. All authors contributed to interpretation of results, editing of the manuscript and approved the final manuscript

## Pre-publication history

The pre-publication history for this paper can be accessed here:

http://www.biomedcentral.com/1741-7015/10/166/prepub
